# Control strategy for connected automated vehicles to reduce car-following risks and energy consumption on foggy highway

**DOI:** 10.1371/journal.pone.0326118

**Published:** 2025-07-03

**Authors:** Rui Chen, Xiaolei He

**Affiliations:** 1 China Huaneng Group Co., Ltd., Beijing, China; 2 Jiangsu Huaneng Smart Energy Supply Chain Technology Co., Ltd., Nanjing, China; 3 China Energy Trading Group Co., Ltd., Xi’an, China; Tongji University, CHINA

## Abstract

The foggy environment negatively affects car-following behavior, increasing rear-end collisions and energy consumption (including fuel consumption and traffic emissions). With advancements in technologies, connected automated vehicles (CAVs) are gradually replacing human-driven vehicles (HDVs) and becoming an integral part of transportation systems. The advent of CAVs offers a new approach to reducing car-following risks and energy consumption in foggy conditions. This study develops a fog-adaptive control framework for CAVs in foggy weather to mitigate car-following risks and reduce energy consumption. First, a foggy-weather car-following model, calibrated using driving simulator data, was selected to describe the behavior of HDVs in foggy highway conditions. Then, based on the model predictive control (MPC) theory, a CAV control strategy was proposed to minimize car-following risks and energy consumption in foggy weather. Finally, a simulation-based verification paradigm was established to assess objectives of risk reduction and energy saving under the proposed CAV strategy in mixed traffic. The results show that car-following risks and energy consumption vary under different fog densities and speed limit conditions. The proposed CAV control strategy can effectively reduce car-following risks by suppressing speed fluctuations, thereby lowering energy consumption in foggy mixed vehicular streams. At a 100% CAV penetration rate, the average reductions in various scenarios of fog density and speed limit conditions are as follows: ITC by 80.74%, DRAC by 59.44%, fuel consumption by 27.62%, CO_2_ emissions by 27.62%, CO emissions by 9.57%, HC emissions by 6.21%, and NOx emissions by 11.55%.

## 1. Introduction

With the rapid growth of global motor vehicle ownership, issues related to traffic safety and energy consumption, including fuel consumption and traffic emissions, have become increasingly prominent. A five-year highway observation study in Indiana revealed that, out of 338 observed highway segments, only 120 segments experienced no traffic collisions, while the remaining 218 segments recorded a total of 5,737 traffic accidents [1]. According to statistics, road transport injuries account for approximately 1.24 million cause 1.24 million fatalities per annum globally, and an additional 20–50 million people are injured, leading to economic losses exceeding $500 billion [2]. At the same time, the large-scale operation of motor vehicles results in significant fuel consumption and severe environmental pollution [3,4]. For example, annual gasoline consumption in the United States approximates 143 billion gallons, translating to a daily demand of 391 million gallons [5]. The problems of highway traffic safety and energy consumption are fundamentally rooted in car-following behavior. Therefore, it is crucial to analyze the risks associated with car-following behavior and explore effective strategies to reduce energy consumption, as these are essential for enhancing the sustainability and safety of transportation systems.

Rear-end collisions constitute the predominant accident modality of accident on highways [6]. Low visibility in foggy weather hinders drivers’ ability to observe the movements of multiple vehicles ahead, increasing their reaction time [7]. If the car-following distance is too short under these conditions, drivers must react more quickly and often tend to brake more forcefully to avoid a collision, which increases the overall speed variation within the traffic flow and makes rear-end collisions more likely [8]. In addition, Peng et al. [9] conducted a study by collecting real-world driving data to investigate the effects of fog on driving behavior and traffic parameters. The results indicated that the impact of fog on traffic varies by vehicle type, lane, and visibility conditions. Compared to trucks, the effects of fog are more pronounced on passenger vehicles, and the impact on traffic in inner lanes is more significant than on outer lanes. Significant differences were found in the average headway, vehicle speed, and headway standard deviation under different visibility levels.

Furthermore, fog, as a common adverse weather phenomenon, interacts with energy consumption. Fog is typically formed when pollutants such as nitrogen oxides and particulate matter from vehicle exhausts accumulate in the atmosphere and combine with water vapor, significantly reducing visibility [5,10]. To adapt to this harsh environment, drivers usually reduce speed or engage in more aggressive braking, leading to speed fluctuations that increase fuel consumption and result in higher emissions of pollutants [5,11]. Then it will lead to a vicious cycle between pollutant emissions and the foggy environment. Pollutant emissions contribute to the formation of fog, and the presence of fog further exacerbates fuel consumption and increases the emission of pollutants.

In recent years, the study of connected automated vehicles (CAVs) platoon control strategies has emerged as a focal point in intelligent transportation research [12–18]. Early studies on reducing fuel consumption and emissions mainly employed traditional control methods for car-following. For instance, Peppard [19] proposed a longitudinal motion controller based on proportion integration differentiation (PID) control, which adjusts throttle openings in real-time to track the inter-vehicle distance. However, this approach struggled to effectively coordinate the multi-objective optimization of energy consumption and emissions. Cai et al. [20] developed a Fuzzy Radial Basis Function Network (FRBFN) model, which, although not requiring training data or a vehicle’s longitudinal dynamics model, still demonstrated certain advantages over traditional PID control. However, it lacked consideration of emissions. The model predictive control (MPC) has become the mainstream approach for CAV car-following control due to its rolling optimization mechanism and explicit constraint handling capability [21–24]. Some studies have also incorporated energy consumption into MPC-based control objectives, which not only fulfill control tasks but also reduce fuel consumption to lower traffic emissions [25–27]. For example, Ruan et al. [28] established a finite-time optimal control problem for car-following and energy management strategies (CFTOC), and simulation results showed that the proposed controller could improve energy efficiency, driving safety, and comfort. He et al. [29] proposed an MPC-based longitudinal control strategy that considers energy consumption, using an efficient energy management strategy (EMS) to optimize torque distribution. The results showed that the strategy improved speed tracking accuracy by 58.93%, increased powertrain efficiency by 40.93%, and reduced equivalent energy consumption by 9.29%. Hu et al. [30] developed an MPC-based multi-objective control framework for car-following scenarios, optimizing vehicle speed and engine torque to improve fuel economy and reduce exhaust emissions. Their control strategy resulted in reductions of 10.49% in fuel consumption, 48.02% in CO emissions, and 55.38% in HC and NOx emissions.

To reduce car-following risks on highways, especially under low visibility in foggy conditions, three main approaches are currently employed. The first is speed limit control on highways during foggy conditions. Lowering the speed limit on highways can significantly reduce the risk of rear-end collisions in foggy weather [8]. Additionally, the speed limit can be dynamically adjusted based on visibility or inter-vehicle distance, enhancing the adaptability of this control method [31,32]. The second approach is improving the car-following strategy for CAVs in foggy conditions. For example, Han et al. proposed an adaptive automatic emergency braking model, which performs better than traditional models in foggy environments [33]. Das and Ahmed adjusted car-following model parameters based on natural driving data, resulting in improved performance under foggy conditions [34]. Based on queue control and CAV lane management, Peng et al. [35] developed a hybrid traffic flow control framework and evaluated its impact on traffic oscillations and safety in mixed traffic flow. The results indicated that the proposed framework effectively mitigates traffic oscillations, reduces the propagation of shockwaves, and lowers collision risks. The third approach involves proactive prediction, where meteorological data is used to predict potential low-visibility areas on highways in the short term. This prediction method can be integrated with vehicle navigation systems to optimize vehicle routing and alert drivers to upcoming risks [4]. These measures demonstrate effective mitigation of fog-related rear-end collisions risks while enhancing safety in vehicular operations.

From the above analysis, it is evident that previous literature has proposed a series of CAV control methods aimed at reducing rear-end collision risks and energy consumption on highways. However, there are several limitations in the existing research: 1) While some studies have explored the impact of fog on traffic safety in depth, few have comprehensively analyzed the joint effects of fog density and speed limit conditions on car-following risks. Since highways often operate under varying speed limits, it is necessary to analyze car-following risks under different fog densities and speed limits. 2) There is limited research on how fog affects fuel consumption and emissions. Given the global importance of fuel usage and exhaust emissions in transportation, analyzing their characteristics under different fog densities and speed limits is crucial. 3) Although various CAV control strategies have been developed to reduce car-following risks and energy consumption, most of these strategies are designed for clear weather conditions. The optimization of CAVs to reduce car-following risks and energy consumption under foggy conditions has received insufficient attention.

To address these gaps, this paper aims to analyze the effects of different fog densities and speed limit conditions on car-following risks and energy consumption. It also introduces a control strategy for CAVs adapted to foggy conditions, with the goal of reducing car-following risks and energy consumption, which includes fuel consumption and traffic emissions.

The document architecture proceeds through the following organizational logic. Section 2 selects a foggy-weather car-following model derived from driving simulator data to depict the car-following behavior of HDVs in foggy conditions. Section 3 proposes a CAV car-following control strategy. The presented CAV control strategy was validated in reducing car-following risks and energy consumption through numerical simulation experiments in Section 4. Section 5 concludes the paper.

## 2. Car-following model of HDVs

In this section, we select a foggy-weather car-following model based on driving simulator data calibration parameters to characterize the car-following behavior of HDVs in foggy highway environments. Huang et al. [36] calibrated the intelligent driver model (IDM) based on driving simulator data, considering both fog density and speed limits. The results indicated that the calibrated IDM model effectively captures car-following behavior in foggy conditions. Therefore, this study adopts the calibrated IDM model as the car-following model, and the specific expression is as follows:


$$a_{n}(t)=a_{\max}\left[1-{\left(\frac{v_{n}(t)}{V_{\max}}\right)}^{\delta }-{\left(\frac{s_{0}+s_{1}\sqrt{\frac{v_{n}(t)}{V_{\max}}}+Tv_{n}(t)-\frac{v_{n}(t)\Delta v_{n}(t)}{2\sqrt{a_{\max}b}}}{h_{n}(t)-l}\right)}^{2}\right]$$ (1)

where *a*_*n*_(*t*) represents acceleration, *v*_*n*_(*t*) is speed, Δ*v*_*n*_(*t*) is the speed difference, *a*_max_ denotes the maximum acceleration, *δ* is the acceleration parameter, *h*_*n*_(*t*) is the spacing of vehicle *n*, *s*_0_ is the minimum distance gap, *s*_1_ the distance parameter, *T* is the desired time headway, *b* the desired deceleration, *V*_max_ is the maximum speed limit, *l* is the vehicle length, taken as 5 m. The calibration results for the IDM model in foggy scenarios are shown in Table 1 (Huang et al., 2024).

**Table 1:** Calibration results of IDM model parameters in foggy weather.

Fog densities	Speed limits (km/h)	*a*_max_ (m/s^2^)	*b* (m/s^2^)	*T* (s)	*δ*	*s*_0_ (m)	*s*_1_ (m)
Light fog	40	5.780	1.055	0.542	4.879	0.129	0.309
	60	5.923	4.367	0.517	4.825	2.056	0.883
	80	5.891	4.273	0.606	4.885	1.229	0.874
	100	5.932	2.829	0.586	4.923	0.875	0.905
Heavy fog	40	5.838	3.898	0.516	4.846	1.180	0.395
	60	5.831	3.708	0.543	4.884	1.481	1.300
	80	5.958	3.684	0.528	4.778	0.532	0.466
	100	5.923	3.672	0.590	4.908	1.899	0.729

It should be noted that fog conditions significantly influence car-following behavior. The previous studies [8,36,37] demonstrate a positive correlation between visibility and both spacing headway and time headway. Specifically, as visibility improves in fog, behavioral heterogeneity, i.e., encompassing intra-driver variability and inter-driver differences, tends to increase, alongside a rise in the average following distance. The parameter values listed in Table 1 collectively govern car-following dynamics under foggy conditions, primarily manifested in adjustments to spacing headway and time headway.

Furthermore, as observed in Table 1, some parameters in the dense fog scenario are lower than those in the light fog scenario. This is because, in dense fog, visibility is significantly reduced, and drivers’ ability to perceive changes in relative speed and inter-vehicle distance becomes less responsive. As a result, the sensitivity parameters decrease further, leading to certain calibration values being lower under specific speed limit conditions compared to the light fog scenario. Additionally, in dense fog, drivers experience increased psychological stress, which may limit the precision of their responses and the extent to which they can adjust parameters, causing some parameter values to be smaller than those observed in light fog conditions. It is important to note that the dynamic expression of car-following behavior is inherently a system involving the coupling of multiple parameters. Therefore, changes in individual parameters under dense fog conditions can still be compensated through dynamic interactions between parameters, thereby maintaining the overall stability of the behavior pattern.

## 3. Control strategy for CAVs

**Fig 1:**
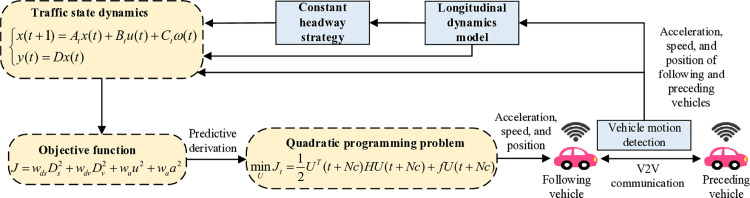
Flowchart of the proposed CAV control strategy.

In this section, we present a CAV control strategy in foggy weather based on the model predict control theory. Regarding MPC formulation, it is necessary to establish the vehicle state equations, define the objective function, and then transform the objective function into a standard quadratic programming problem for solution [38]. Following this control framework, the control process of the proposed CAV control strategy is shown in Fig 1. First, based on V2V communication technology, the acceleration, speed, and position information of both the lead and following vehicles are obtained. Using this data, the error between the actual and desired spacing, as well as the speed difference, is calculated. These, along with the following vehicle’s acceleration, are combined as system state variables to form the vehicle state equations. Next, an objective function is defined, incorporating safety tracking and energy consumption minimization. The objective function over the prediction horizon is derived, transformed into a standard quadratic programming problem, and solved. The final output consists of the vehicle’s acceleration, speed, and position.

### 3.1 Traffic state dynamics

In the car-following state, ensuring safety depends on maintaining an appropriate distance from the preceding vehicle. Therefore, maintaining a safe distance between the following vehicle and the preceding vehicle is one of the primary objectives of the CAV control strategy. In this paper, the constant headway strategy is adopted to obtain desired distance for CAVs, which is calculated as follows:


$$d_{i}^{des}(t)=d_{0}+h_{i}v_{i}(t)$$ (2)

where $d_{i}^{des}(t)$ is desired distance, *d*_0_ is the minimum distance between the following and preceding vehicles at stop, set to 2m [39], *h*_*i*_ represents the CAV headway, taken as 2.2s [40], and *v*_*i*_(*t*) is speed of following vehicle.

For a first-order inertial system, the longitudinal dynamics model can be expressed as:


$$\{\begin{array}{c} {}*20l{\dot{s}}_{i}(t)=v_{i}(t)\\ {\dot{v}}_{i}(t)=a_{i}(t)\\ {\dot{a}}_{i}(t)=\frac{K(u_{i}(t)-a_{i}(t))}{\lambda } \end{array}$$ (3)

where ${\dot{s}}_{i}(t)$ is the first-order derivative of distance of following vehicle with respect to time, ${\dot{v}}_{i}(t)$ is the first-order derivative of speed of following vehicle with respect to time, ${\dot{a}}_{i}(t)$ is the first-order derivative of acceleration of following vehicle with respect to time, *s*_*i*_(*t*) is distance of following vehicle, *a*_*i*_(*t*) is following vehicle’s acceleration, *K* is system gain, taken as 1, and *λ* is time constant, taken as 0.01s.

Based on the vehicle longitudinal dynamics model, the spacing error, i.e., difference between actual and desired spacing, can be calculated as follows:


$$D_{s}=\left(s_{i-1}-s_{i}\right)-(d_{i}^{des}+l)$$ (4)

where *D*_*s*_ represents spacing error.

Also, the speed difference can be calculated as follows:


$$D_{v}=v_{i-1}-v_{i}$$ (5)

where *D*_*v*_ represents speed difference.

Previous MPC strategies for clear-weather environments typically use position, speed, and acceleration as state variables [41–43]. However, position and speed primarily describe the kinematic characteristics of individual vehicles and do not directly capture the dynamic interaction between the lead and following vehicles. In low-visibility conditions such as fog, vehicle-to-vehicle (V2V) communication enables the following vehicle to obtain information about the lead vehicle’s motion, allowing for the calculation of the spacing error and speed difference between the two vehicles. Therefore, this study proposes using spacing error as a state variable in the MPC control strategy, as it directly quantifies the safety margin between the vehicles. Additionally, incorporating the speed difference as a state variable allows for the representation of the collision risk trend, providing a more accurate depiction of the relative motion between the lead and following vehicles.

Hence, the purpose of the proposed CAV control strategy is to ensure that the spacing error, speed difference, and acceleration converge to zero. Therefore, the spacing error, speed difference, and acceleration of following vehicle are selected as predictive model indicators, while the preceding vehicle’s acceleration is considered the system perturbation input. The continuous-state equation of the vehicle is then established as follows:


$$\dot{x}(t)=Ax(t)+Bu(t)+Cw(t)$$ (6)

where *x*(*t*) represents state vector of vehicle, taken as $x(t)={\left[\begin{array}{ccc} {}*20cD_{s} & D_{v} & a \end{array}\right]}^{T}$, $\dot{x}(t)$ represents the first-order derivative of state vector *x*(*t*) refers to time *t*, i.e., $\dot{x}(t)=\left[\begin{array}{ccc} {}*20cv_{i-1}-v_{i} & a_{i-1}-a_{i} & \frac{K(u_{i}(t)-a_{i}(t))}{\lambda } \end{array}\right]$, *u*(*t*) represents system input, i.e., the desired acceleration of following vehicle, *w*(*t*) represents system disturbance, i.e., acceleration of the preceding vehicle. *A*, *B*, and *C* represent system state coefficient matrix, system input coefficient matrix, and system disturbance coefficient matrix, respectively. Each coefficient matrix is computed as follows:


$$A=\left[\begin{array}{ccc} {}*20c0 & 1 & -h_{i}\\ 0 & 0 & -1\\ 0 & 0 & -\frac{1}{\lambda } \end{array}\right],B=\left[\begin{array}{c} {}*20c0\\ 0\\ \frac{1}{\lambda } \end{array}\right],C=\left[\begin{array}{c} {}*20c0\\ 1\\ 0 \end{array}\right]$$ (7)

Note that Eq. (6) is the continuous-state vector of vehicle, however, in the optimal control scheme, a discrete-state vector is required for iteration and prediction processes. Therefore, it is necessary to discretize Eq. (6) to obtain the discrete-state vector that suitable for numerical computation.

Using the forward Euler method, Eq. (6) can be expressed as follows:


$$\dot{x}(t)=\frac{x(t+1)-x(t)}{T}=Ax(t)+Bu(t)+C\omega (t)$$ (8)

where *T* represents sampling period.

We can rewrite Eq. (8) as follows:


x(t+1)=(I+AT)x(t)+BTu(t)+CTω(t) (9)

where *I* is the identity matrix, Let *I*+*AT*=*A*_*t*_, *BT* = *B*_*t*_, and *CT* = *C*_*t*_. Then, Eq. (9) can be expressed as:


$$x(t+1)=A_{t}x(t)+B_{t}u(t)+C_{t}\omega (t)$$ (10)

where,


$$A_{t}=\left[\begin{array}{ccc} {}*20c1 & T & -h_{i}T\\ 0 & 1 & -T\\ 0 & 0 & 1-\frac{1}{\lambda }T \end{array}\right],B_{t}=\left[\begin{array}{c} {}*20c0\\ 0\\ \frac{1}{\lambda }T \end{array}\right],C_{t}=\left[\begin{array}{c} {}*20c0\\ T\\ 0 \end{array}\right]$$ (11)

The system output can be designed as follow:


y(t)=Dx(t) (12)

where *y*(*t*) represents the system output, and *D* represents the system output coefficient matrix, as follows:


$$D=\left[\begin{array}{ccc} {}*20c1 & 0 & 0\\ 0 & 1 & 0\\ 0 & 0 & 1 \end{array}\right]$$ (13)

Combining Eq. (9) and Eq. (12), the discrete-state vector can be obtained as follows:


$$\left\{ \begin{array}{c} x(t+1)=A_{t}x(t)+B_{t}u(t)+C_{t}\omega (t)\\ y(t)=Dx(t) \end{array}\right.$$ (14)

We can know that from Eq. (14), if the state vector, system inputs and system disturbances at time *t* are known, the state vector and system outputs at time *t* + 1 can be calculated. In order to make the designed MPC model predict future states and control variables, setting the prediction horizon as *Np*, while control horizon as *Nc*. Then the state vector in *Np* can be calculated as follows:


$$\left\{ \begin{array}{c} x(t+1)=A_{t}x(t)+B_{t}u(t)+C_{t}w(t)\\ x(t+2)=A_{t}^{2}x(t)+A_{t}B_{t}u(t)+A_{t}^{0}B_{t}u(t+1)+\\ A_{t}C_{t}w(t)+A_{t}^{0}C_{t}w(t+1)\\ x(t+3)=A_{t}^{3}x(t)+A_{t}^{2}B_{t}u(t)+A_{t}B_{t}u(t+1)+A_{t}^{0}B_{t}u(t+2)+\\ A_{t}^{2}C_{t}w(t)+A_{t}C_{t}w(t+1)++A_{t}^{0}C_{t}w(t+2)\\ \vdots \\ x(t+Nc)=A_{t}^{Nc}x(t)+A_{t}^{Nc-1}B_{t}u(t)+\ldots A_{t}^{0}B_{t}u(t+Nc-1)+\\ A_{t}^{Nc-1}C_{t}w(t)+\ldots A_{t}^{0}C_{t}w(t+Nc-1)\\ \vdots \\ x(t+Np)=A_{t}^{Np}x(t)+A_{t}^{Np-1}B_{t}u(t)+\ldots A_{t}^{0}B_{t}u(t+N_{p}-1)+\\ A_{t}^{Np-1}C_{t}u(t)+\ldots A_{t}^{0}C_{t}u(t+N_{p}-1) \end{array}\right.$$ (15)

Similarly, the system output in *Np* can be calculated as follows:


$$\left\{ \begin{array}{c} y(t)=Dx(t)\\ y(t+1)=DA_{t}x(t)+DB_{t}u(t)+DC_{t}w(t)\\ y(t+2)=DA_{t}^{2}x(t)+DA_{t}B_{t}u(t)+DA_{t}^{0}B_{t}u(t+1)+DA_{t}C_{t}w(t)+DA_{t}^{0}C_{t}w(t+1)\\ y(t+3)=DA_{t}^{3}x(t)+DA_{t}^{2}B_{t}u(t)+DA_{t}B_{t}u(t+1)+DA_{t}^{0}B_{t}u(t+2)+\\ DA_{t}^{2}C_{t}w(t)+DA_{t}C_{t}w(t+1)+DA_{t}^{0}C_{t}w(t+2)\\ \vdots \\ y(k+Nc)=DA_{t}^{Nc}x(t)+DA_{t}^{Nc-1}B_{t}u(t)+\ldots +\\ DA_{t}^{0}B_{t}u(k+Nc-1)+DA_{t}^{Nc-1}C_{t}w(t)+\ldots DA_{t}^{0}C_{t}w(k+Nc-1)\\ \vdots \\ y(k+Np)=DA_{t}^{Np}x(t)+DA_{t}^{Np-1}B_{t}u(t)+\ldots +\\ DA_{t}^{0}B_{t}u(t+Np-1)+DA_{t}^{Np-1}C_{t}w(t)+\ldots DA_{t}^{0}C_{t}w(t+Np-1) \end{array}\right.$$ (16)

Rewriting Eq. (15) yields:


$$X(t+Np)=A_{x}x(t)+B_{x}U(t+Nc)+C_{x}W(t+Np)$$ (17)

where,


$$\begin{array}{cc} & X(t+Np)=\left[\begin{array}{c} x(t+1)\\ x(t+2)\\ \vdots \\ x(t+Np) \end{array}\right],U(t+Nc)=\left[\begin{array}{c} u(t)\\ u(t+1)\\ \vdots \\ u(t+Nc) \end{array}\right],W(t+Np)=\left[\begin{array}{c} w(t)\\ w(t+1)\\ \vdots \\ w(t+Np) \end{array}\right],\\ & A_{x}=\left[\begin{array}{c} A_{t}\\ A_{t}^{2}\\ \vdots \\ A_{t}^{Np} \end{array}\right],B_{x}=\left[\begin{array}{cccc} B_{t} & 0 & \cdots & 0\\ A_{t}B_{t} & B_{t} & \cdots & 0\\ \vdots & \cdots & \cdots & \vdots \\ A_{t}^{Nc-1}B_{t} & A_{t}^{Nc-2}B_{t} & \cdots & B_{t}\\ A_{t}^{Nc}B_{t} & A_{t}^{Nc-1}B_{t} & \cdots & A_{t}B_{t}\\ \vdots & \vdots & \vdots & \vdots \\ A_{t}^{Np-1}B_{t} & A_{t}^{Np-2}B_{t} & \cdots & A_{t}^{Np-Nc-1}B_{t} \end{array}\right]\\ & C_{x}=\left[\begin{array}{cccc} C_{t} & 0 & \cdots & 0\\ A_{t}C_{t} & C_{t} & \cdots & 0\\ \vdots & \cdots & \cdots & \vdots \\ A_{t}^{Nc-1}C_{t} & A_{t}^{Nc-2}C_{t} & \cdots & C_{t}\\ A_{t}^{Nc}C_{t} & A_{t}^{Nc-1}C_{t} & \cdots & A_{t}C_{t}\\ \vdots & \vdots & \vdots & \vdots \\ A_{t}^{Np-1}C_{t} & A_{t}^{Np-2}C_{t} & \cdots & A_{t}^{Np-Nc-1}C_{t} \end{array}\right] \end{array}$$ (18)

Rewriting Eq. (16) yields:


$$Y(t+Np)=A_{y}x(t)+B_{y}U(t+Nc)+C_{y}W(t+Np)$$ (19)

where,


$$\begin{array}{c} Y(t+Np)=\left[\begin{array}{c} y(t+1)\\ y(t+2)\\ \vdots \\ y(t+Np) \end{array}\right],B_{y}=\left[\begin{array}{cccc} {}*20cDB_{t} & 0 & \cdots & 0\\ DA_{t}B_{t} & DB_{t} & \cdots & 0\\ \vdots & \cdots & \cdots & \vdots \\ DA_{t}^{Nc-1}B_{t} & DA_{t}^{Nc-2}B_{t} & \cdots & DB_{t}\\ DA_{t}^{Nc}B_{t} & DA_{t}^{Nc-1}B_{t} & \cdots & DA_{t}B_{t}\\ \vdots & \vdots & \vdots & \vdots \\ DA_{t}^{Np-1}B_{t} & DA_{t}^{Np-2}B_{t} & \cdots & DA_{t}^{Np-Nc-1}B_{t} \end{array}\right]\\ A_{y}=\left[\begin{array}{c} DA_{t}\\ DA_{t}^{2}\\ \vdots \\ DA_{t}^{Np} \end{array}\right],C_{y}=\left[\begin{array}{cccc} {}*20cDC_{t} & 0 & \cdots & 0\\ DA_{t}C_{t} & DC_{t} & \cdots & 0\\ \vdots & \cdots & \cdots & \vdots \\ DA_{t}^{Nc-1}C_{t} & DA_{t}^{Nc-2}C_{t} & \cdots & DC_{t}\\ DA_{t}^{Nc}C_{t} & DA_{t}^{Nc-1}C_{t} & \cdots & DA_{t}C_{t}\\ \vdots & \vdots & \vdots & \vdots \\ DA_{t}^{Np-1}C_{t} & DA_{t}^{Np-2}C_{t} & \cdots & DA_{t}^{Np-Nc-1}C_{t} \end{array}\right] \end{array}$$ (20)

### 3.2 Objective function

As we mentioned before, the purpose of the proposed CAV control strategy is to make the spacing error and speed difference tends to zero. Meanwhile, reducing car-following risks and energy consumption is also an objective with the CAV control strategy. Relevant studies have shown that car-following risks and energy consumption are closely related to acceleration. It means that higher acceleration results in increased car-following risks and energy consumption [44].

Consider the objectives outlined above, the objective function of the CAV control strategy can be constructed as follows:


$$J=w_{ds}D_{s}^{2}+w_{dv}D_{v}^{2}+w_{u}u^{2}+w_{a}a^{2}$$ (21)

where *J* represents system objective function, *w*_*ds*_ represents spacing error weight coefficient, *w*_*dv*_ represents speed difference weight coefficient, *w*_*a*_ represents acceleration weight coefficient, and *w*_*u*_ represents the desired acceleration weight coefficient.

From Eq. (21), the objective function formulated in this study is directly linked to the defined state variables, incorporating spacing error, speed difference, and acceleration. This design enhances the computational efficiency of the system. Crucially, the spacing error and speed difference in the objective function reflect the system’s safety tracking performance, while the acceleration term accounts for energy consumption and emission economy. Thus, compared to conventional control strategies, our proposed objective function simultaneously addresses both safety tracking and energy-emission efficiency during vehicle operations.

The objective function equation in the prediction horizon can be expressed as:


$$\begin{array}{cc} J_{t} & =\sum_{i=1}^{Np}{\left\|y(t+i)\right\|}_{Q}^{2}+\sum_{i=0}^{Nc}{\left\|u(t+i)\right\|}_{w_{u}}^{2}\\ & ={\left[\begin{array}{c} y(t+1)\\ y(t+2)\\ \vdots \\ y(t+Np) \end{array}\right]}^{T}\left[\begin{array}{cccc} {}*20cQ & & & \\ & Q & & \\ & & \ddots & \\ & & & Q \end{array}\right]\left[\begin{array}{c} y(t+1)\\ y(t+2)\\ \vdots \\ y(t+Np) \end{array}\right]\\ & +{\left[\begin{array}{c} u(t)\\ u(t+1)\\ \vdots \\ u(t+Nc) \end{array}\right]}^{T}\left[\begin{array}{cccc} {}*20lw_{u} & & & \\ & w_{u} & & \\ & & \ddots & \\ & & & w_{u} \end{array}\right]\left[\begin{array}{c} u(t)\\ u(t+1)\\ \vdots \\ u(t+Nc) \end{array}\right]\\ & \mathrm{\backslash vspace}6pt=Y^{T}(t+Np)Q_{Q}Y(t+N_{p})+U^{T}(t+N_{c}){\bar{w}}_{u}U(t+N_{c}) \end{array}$$ (22)

where,


$$Q=\left[\begin{array}{ccc} {}*20cw_{ds} & 0 & 0\\ 0 & w_{dv} & 0\\ 0 & 0 & w_{a} \end{array}\right],\;Q_{Q}=\left[\begin{array}{cccc} {}*20cQ & & & \\ & Q & & \\ & & \ddots & \\ & & & Q \end{array}\right],{\bar{w}}_{u}=\left[\begin{array}{cccc} {}*20cw_{u} & & & \\ & w_{u} & & \\ & & \ddots & \\ & & & w_{u} \end{array}\right]$$ (23)

We can rewrite Eq. (22) as follows:


$$J_{t}=Y^{T}(t+Np)Q_{Q}Y(t+N_{p})+U^{T}(t+N_{c}){\bar{w}}_{u}U(t+N_{c})$$ (24)

Substituting Eq. (19) into Eq. (23) yields:


$$\begin{array}{cc} J_{t} & =Y^{T}(t+Np)Q_{Q}Y(t+N_{p})+U^{T}(t+N_{c}){\bar{w}}_{u}U(t+N_{c})\\ & ={\left[A_{y}x(t)+B_{y}U(t+N_{c})+C_{y}W(t+N_{p})\right]}^{T}Q_{Q}\times \\ & \left[A_{y}x(t)+B_{y}U(t+N_{c})+C_{y}W(t+N_{p})\right]+U^{T}(t+N_{c}){\bar{w}}_{u}U(t+N_{c})\\ & =\left[x^{T}(t)A_{y}^{T}Q_{Q}+U^{T}(t+N_{c})B_{y}^{T}Q_{Q}+W^{T}(t+N_{p})C_{y}^{T}Q_{Q}\right]\times \\ & \left[A_{y}x(t)+B_{y}U(t+N_{c})+C_{y}W(t+N_{p})\right]+U^{T}(t+N_{c}){\bar{w}}_{u}U(t+N_{c})\\ & =x^{T}(t)A_{y}^{T}Q_{Q}A_{y}x(t)+U^{T}(t+N_{c})B_{y}^{T}Q_{Q}A_{y}x(t)+\\ & W^{T}(t+N_{p})C_{y}^{T}Q_{Q}A_{y}x(t)+x^{T}(t)A_{y}^{T}Q_{Q}B_{y}U(t+N_{c})+\\ & U^{T}(t+N_{c})B_{y}^{T}Q_{Q}B_{y}U(t+N_{c})+W^{T}(t+N_{p})C_{y}^{T}Q_{Q}B_{y}U(t+N_{c})+\\ & x^{T}(t)A_{y}^{T}Q_{Q}C_{y}W(t+N_{p})+U^{T}(t+N_{c})B_{y}^{T}Q_{Q}C_{y}W(t+N_{p})+\\ & W^{T}(t+N_{p})C_{y}^{T}Q_{Q}C_{y}W(t+N_{p})+U^{T}(t+N_{c}){\bar{w}}_{u}U(t+N_{c}) \end{array}$$ (25)

Deleting the terms that do not affect the objective function, we can obtain that:


$$\begin{array}{cc} J_{t}= & \;\;U^{T}(t+Nc)\left[B_{y}^{T}Q_{Q}B_{y}+{\bar{w}}_{u}\right]U(t+Nc)\\ & +2\left[x^{T}(t)A_{y}^{T}Q_{Q}B_{y}+W^{T}(t+Np)C_{y}^{T}Q_{Q}B_{y}\right]U(t+Nc) \end{array}$$ (26)

Let:


$$\left\{ \begin{array}{c} H=B_{y}^{T}Q_{Q}B_{y}+{\bar{w}}_{u}\\ f=B_{y}^{T}Q_{Q}A_{y}x(t)+B_{y}^{T}Q_{Q}C_{y}W(t+Np) \end{array}\right.$$ (27)

We can rewrite Eq. (26) as follows:


$$J_{t}=U^{T}(t+Nc)HU(t+Nc)+2fU(t+Nc)$$ (28)

Transforming Eq. (28) into the standard form of the quadratic programming problem, as follows:


$$\underset{U}{\min}J_{t}=\frac{1}{2}U^{T}(t+Nc)HU(t+Nc)+fU(t+Nc)$$ (29)

The optimal output of the system can be derived through quadratic programming solutions, thus get an optimal speed of the following vehicle in next time step. Continuous cycling of this process at each tine step enables dynamic control of the following vehicle.

## 4. Simulation experiments

This section conducts numerical experiments to analyze car-following risks and energy consumption characteristics under foggy conditions. First, we use the foggy-weather HDVs car-following model and the CAVs car-following control strategy to perform numerical simulations and generate vehicle trajectory data. These output trajectory data are then used as input for the rear-end collision and energy consumption surrogate models, allowing us to calculate car-following risks and energy consumption characteristics under various scenarios. In Section 2 and Section 3, we have presented the foggy-weather HDVs car-following model and the CAVs control strategy. In Section 4.1, we will present the simulation scenarios, and in Section 4.2 and Section 4.3, we will analyze car-following risks and energy consumption, respectively.

### 4.1. Experiment scenario

Due to the inherent challenges in analyzing real-world data on car-following risks and energy consumption/emissions under foggy conditions on highways, this study employs numerical simulation to investigate these factors. Multiple studies [9,45,46] have indicated that drivers exhibit more frequent acceleration and deceleration behaviors in foggy weather, which are the primary car-following actions. Therefore, in this section, we set up an acceleration and deceleration simulation scenario to analyze car-following risks and energy consumption of heterogeneous traffic flow on a foggy highway.

The simulation involves a vehicle fleet consisting of a leading vehicle and following vehicles, where CAVs are evenly distributed within the fleet. The number of CAVs and HDVs in the platoon is determined by the MPR. When MPR = 0, it indicates that all following vehicles are HDVs, meaning no control strategy is applied to the vehicles in the platoon. The motion of the lead vehicle is referenced from previous studies, while the motion of the following vehicles is described by either the foggy-weather HDV model or the CAV strategy.

**Fig 2:**
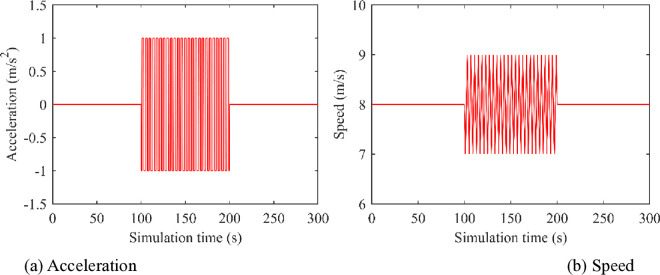
Acceleration and speed curves of the lead vehicle.

The motion trajectory of the leading vehicle is shown as below [47]. At the initial moment, the leading vehicle moves at a constant speed of 8 m/s for 100 seconds. Then, between 100 and 200 seconds, the leading vehicle performs periodic acceleration and deceleration, with each cycle lasting 4 seconds and an acceleration of ±1 m/s². Finally, after 200 seconds, the leading vehicle keeps a constant speed of 8 m/s until the simulation ends. The simulation step size and the total simulation time is set as 0.01s and 300s respectively. To better illustrate the leading vehicle’s motion characteristics, Fig 2 presents its acceleration profile and speed trajectory during the simulation period.

### 4.2 Car-following risk analysis

#### 4.2.1 Surrogate safety measures.

The time-to-collision (TTC) is one of the most universally used indicators for evaluating car-following risks on highways. It is defined as the time gap before a rear-end collision occurs, assuming that the current speed difference between the leading and the following vehicles remains constant. TTC is an effective measure for describing the risk of rear-end collisions on highways, and previous studies have utilized TTC as a traffic safety evaluation metric to analyze the risk of rear-end collisions [48]. The TTC calculation formula is as follows [49]:


$$TTC_{n}(t)=\{\begin{array}{cc} {}*20c\frac{x_{n-1}(t)-x_{n}(t)}{v_{n}(t)-v_{n-1}(t)} & v_{n}(t)>v_{n-1}(t)\\ \infty & v_{n}(t)<v_{n-1}(t) \end{array}$$ (30)

where, *x*_*n*_(*t*) is the location of vehicle *n* at time *t*, *TTC*_*n*_(*t*) is the collision time between the *n*th vehicle and the *n*-1th vehicle at time *t*.

As shown in Eq. (30), TTC is only applicable for evaluating the risk of rear-end collisions when the speed of the leading vehicle is less than that of the following vehicles. However, when speeds of the two vehicles are equal, TTC cannot reflect the potential car-following collision risk. To address this issue, many researchers have used the inverse time-to-collision (ITC) as a substitute for TTC, effectively avoiding situations where TTC changes excessively or becomes undefined. The ITC calculation formula is as follows [50]:


$$ITC_{n}(t)=\{\begin{array}{cc} {}*20c\frac{v_{n}(t)-v_{n-1}(t)}{x_{n-1}(t)-x_{n}(t)} & v_{n}(t)\geq v_{n-1}(t)\\ 0 & v_{n}(t)<v_{n-1}(t) \end{array}$$ (31)

When ITC is greater than 0, it indicates the presence of a car-following risk. Furthermore, the larger value of ITC, the higher the risk of a car-following collision.

Furthermore, the deceleration rate to avoid the crash (DRAC) is used to describe the deceleration that the following vehicle must apply during the car-following process to avoid a collision with the lead vehicle. The calculation formula for DRAC is as follows [51]:


$$DRAC_{n}(t)=\{\begin{array}{cc} {}*20c\frac{{\left(v_{n}(t)-v_{n-1}(t)\right)}^{2}}{2\left(x_{n-1}(t)-x_{n}(t)\right)} & v_{n}(t)\geq v_{n-1}(t)\\ 0 & v_{n}(t)<v_{n-1}(t) \end{array}$$ (32)

When DRAC is greater than 0, it indicates the presence of a car-following collision risk. Additionally, the higher the DRAC value, the greater the car-following risk. Furthermore, if DRAC exceeds the maximum braking deceleration, it means that merely applying deceleration is no longer sufficient to prevent a collision between the two vehicles.

Based on the above discussion, this study selects ITC and DRAC as the car-following risk evaluation metrics for the subsequent analysis of car-following collision risks on foggy highways.

#### 4.2.2 Results of car-following risks.

Based on numerical simulation experiments and safety evaluation indexes, we can calculate car-following collision risk indicators under different scenarios. Since the proposed CAV control strategy is compared with HDVs, we selected CAV market penetration rate (MPR) at 0 as the baseline scenario to calculate the reduction in car-following risk under the proposed CAV control strategy for different scenarios in this section, while fuel consumption and emissions will be compared in Section 4.3. To further demonstrate the effectiveness of the proposed strategy, we will validate its performance in reducing car-following risk and energy consumption under varying fog intensities and speed limit conditions. Additionally, from a vehicle operational mechanism perspective, we conducted an in-depth analysis to reveal the underlying reasons why CAV participation leads to reductions in car-following risk and energy consumption within the platoon.

**Fig 3:**
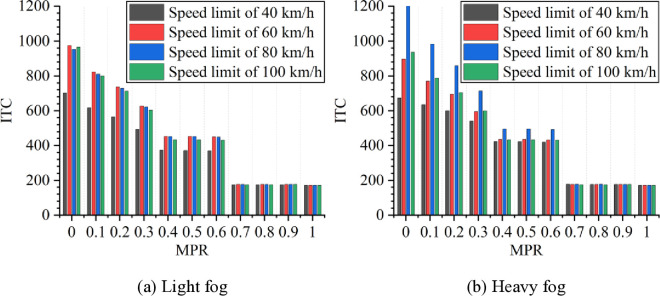
ITC under different conditions.

**Fig 4:**
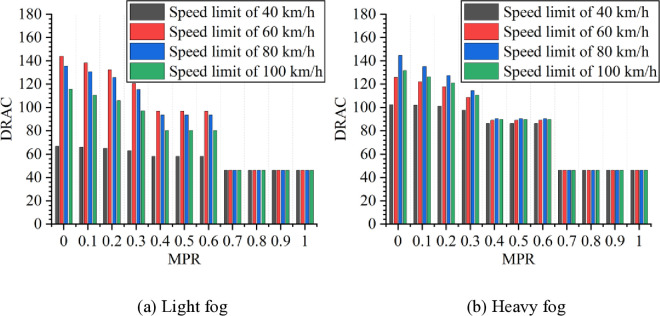
DRAC under different conditions.

As shown in Fig 3 and Fig 4, it can be observed that when the MPR of CAVs is 0, not only the light fog but also the heavy fog shows the highest ITC and DRAC values when speed limit is 60 km/h and 80 km/h, respectively. In the meanwhile, the lowest values of ITC and DRAC occur at a speed limit of 40 km/h. In the light fog scenario, the ITC is higher than in the heavy fog scenario at speed limits of 40, 60, and 100 km/h. However, at a speed limit of 80 km/h, the ITC in the light fog scenario is lower than in the heavy fog scenario. For DRAC, in the light fog scenario, it is higher than in the heavy fog scenario at when speed limit is 60 km/h, while comparing with the light fog scenario, DRAC is lower than in the heavy fog scenario at speed limits of 40, 80, and 100 km/h.

**Fig 5:**
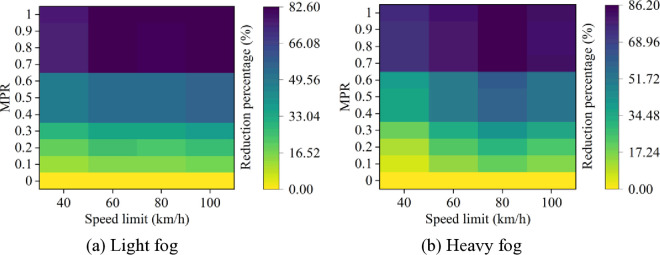
ITC reduction percentages under different conditions.

**Fig 6:**
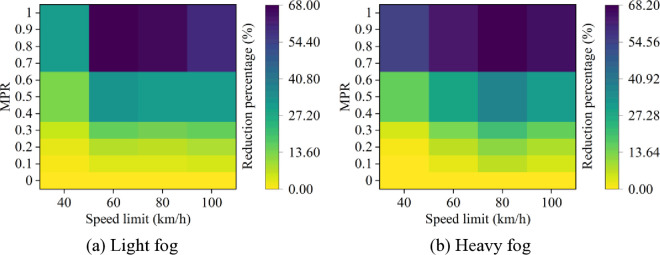
DRAC reduction percentages under different conditions.

Moreover, it can be observed from Fig 3 and Fig 4 that as MPR increases, the rear-end collision risk decreases, indicating that the proposed strategy of CAVs is effective in reducing car-following risks in foggy conditions. Based on the results in Fig 3 and Fig 4, the reduction in car-following risk indicators under different MPR conditions is calculated, with the results shown in Fig 5 and Fig 6. It can be seen that as the MPR increases, the reduction ratio of car-following risks in different scenarios also increases. When MPR = 1, the ITC and DRAC for different scenarios are reduced by an average of 80.74% and 59.44%, respectively.

Additionally, it is evident that under different MPR conditions, the largest reduction in ITC and DRAC occurs when speed limit is 80 km/h and 100 km/h in different scenarios, while the smallest reduction is observed when speed limit is 40 km/h. This suggests that CAVs have more effectiveness in reducing car-following risks in scenarios with higher baseline risk at higher speed limits. Moreover, when MPR ≥ 0.6, the trend of risk reduction starts to flatten, as the car-following behavior becomes primarily controlled by CAVs. In this case, the acceleration and deceleration behaviors within the fleet have already been significantly improved, and the car-following risk is effectively suppressed.

### 4.3 Fuel consumption and emissions analysis

#### 4.3.1 Fuel consumption and emissions model.

To calculate the fuel consumption during the car-following process on foggy highways, this study adopts the fuel consumption model proposed by Kamal et al. [52]. This model, which is based on the vehicle’s instantaneous speed, effectively reflects the fuel consumption characteristics during car-following, and is extensively applied to traffic flow research [53]. The model expression is as follows:


$$f_{n,fuel}(t)=\{\begin{array}{cc} {}*20cb_{0}+b_{1}v_{n}(t)+b_{2}v_{n}^{2}(t)+b_{3}v_{n}^{3}(t)+a_{n}(t)(c_{0}+c_{1}v_{n}(t)+c_{2}v_{n}^{2}(t)) & a_{n}(t)>0\\ b_{0}+b_{1}v_{n}(t)+b_{2}v_{n}^{2}(t)+b_{3}v_{n}^{3}(t) & a_{n}(t)\leq 0 \end{array}$$ (33)

where, *f*_*n,fuel*_(t) is fuel consumption rate of vehicle *n* at time *t*, *b*_0_, *b*_1_, *b*_2_, *b*_3_, *c*_0_, *c*_1_, and *c*_2_ are the model parameters. The values of these parameters are provided in Table 2.

**Table 2:** Parameter values for the fuel consumption model.

Parameters	Values
*b*_0_	0.1569
*b*_1_	2.45 × 10^−2^
*b*_2_	−7.415 × 10^−4^
*b*_3_	5.975 × 10^−5^
*c*_0_	0.07224
*c*_1_	9.681 × 10^−2^
*c*_2_	1.075 × 10^−3^

Relevant studies have shown that fuel consumption and CO_2_ emissions during vehicle operation are strictly linearly related. The CO_2_ emission rate can be calculated using the following relationship:


$$f_{n,CO_{2}}(t)=\delta_{1}v_{n}(t)+\delta_{2}f_{n,fuel}(t)$$ (34)

where *f*_*n, CO2*_(*t*) represents the CO_2_ emission rate of the *n*th vehicle at time *t*, and *f*_*n, fuel*_(*t*) represents the fuel consumption rate of the *n*th vehicle at time *t*. Besides that, *δ*_1_ and *δ*_2_ are model parameters. For gasoline engine vehicles, *δ*_1_ and *δ*_2_ are set to 3.5 × 10^-8 kg^/m and 2.39 kg/l, respectively.

Additionally, this study also considers CO, HC, and NOx emissions during vehicle operation. Fan et al. [54], based on empirical data, grouped the instantaneous emission rates (CO, HC, and NOx) according to the vehicle specific power (VSP) interval (per 1 kW/ton), and then calculated the instantaneous emission rates within each VSP interval unit, obtaining representative traffic instantaneous emission rates for each VSP interval. The VSP calculation formula is as follows:


$$VSP_{n}(t)=v_{n}(t)\times (a_{n}(t)+0.09199)+0.000169v_{n}^{3}(t)$$ (35)

The instantaneous emission rates of CO, HC, and NOx for each VSP interval can be referred from Fan et al. [54].

#### 4.3.2 Results of fuel consumption and emissions.

**Fig 7:**
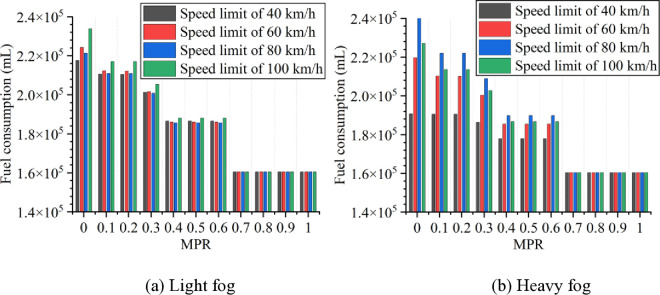
Fuel consumption under different conditions.

**Fig 8:**
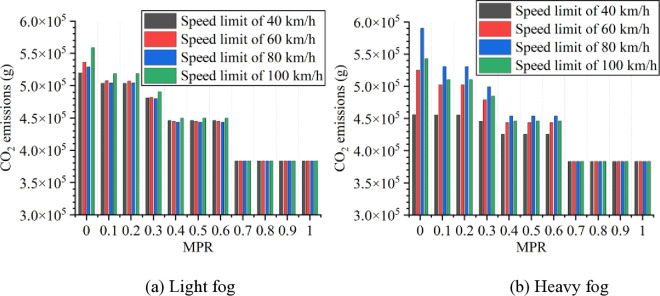
CO_2_ emissions under different conditions.

**Fig 9:**
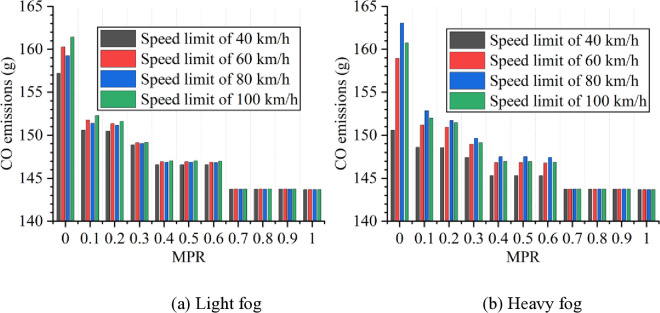
CO emissions under different conditions.

**Fig 10:**
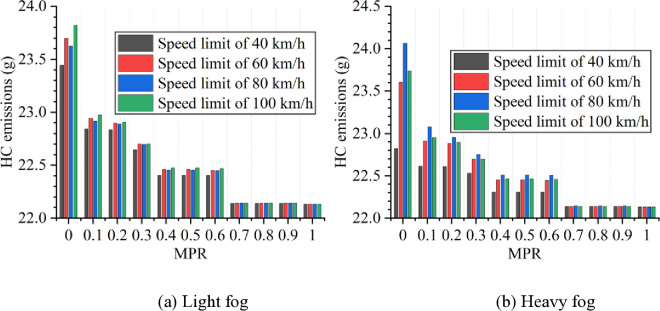
HC emissions under different conditions.

**Fig 11:**
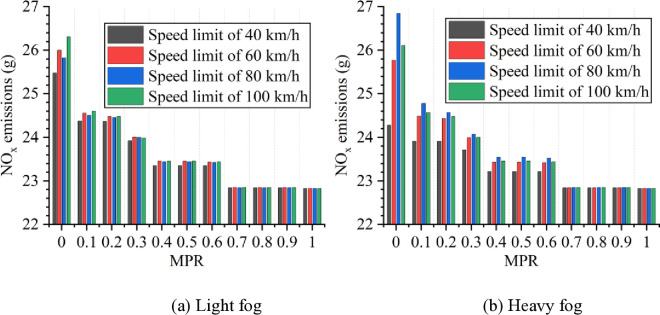
NO_x_ emissions under different conditions.

Based on numerical simulation experiments and the energy consumption models, the results for various scenarios are calculated and presented in Figs 7–11. It is obtained that when MPR = 0, fuel consumption and emissions are highest under light and heavy fog scenarios when speed limit is 100 km/h and 80 km/h, respectively, and are lowest when speed limit is 40 km/h. In scenarios with speed limits of 40, 60, and 100 km/h, fuel consumption and emissions in light fog condition are higher than those in heavy fog, while at a speed limit of 80 km/h, light fog scenario results in lower fuel consumption and emissions than the heavy fog scenario.

**Fig 12:**
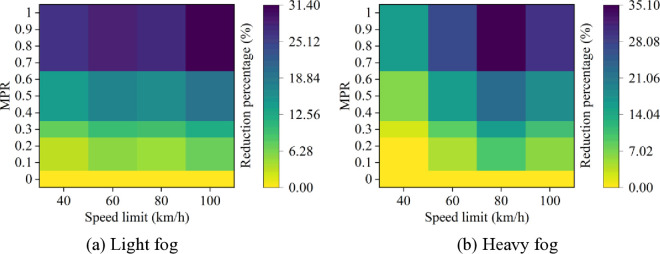
Fuel consumption reduction percentage under different conditions.

**Fig 13:**
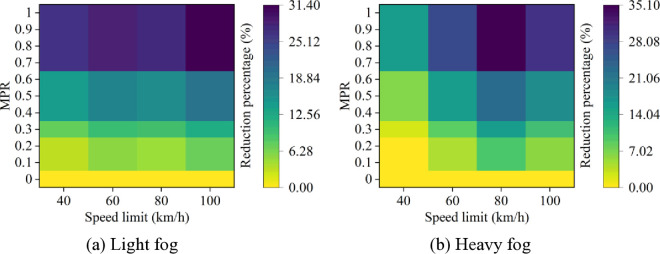
CO_2_ emissions reduction percentage under different conditions.

**Fig 14:**
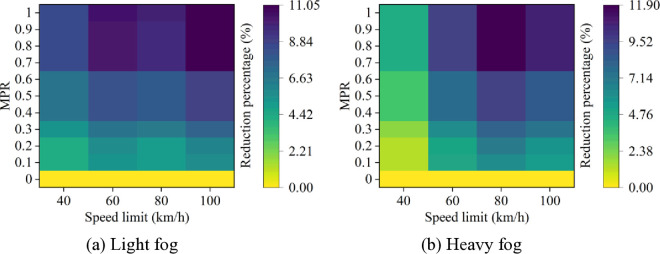
CO emissions reduction percentage under different conditions.

**Fig 15:**
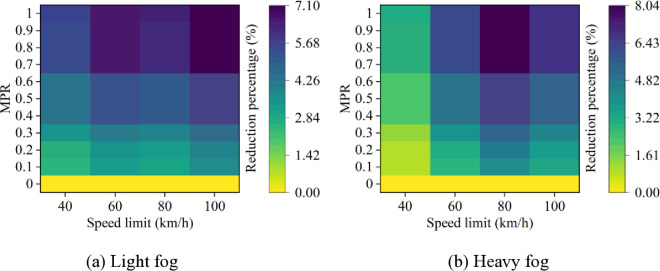
HC emissions reduction percentage under different conditions.

**Fig 16:**
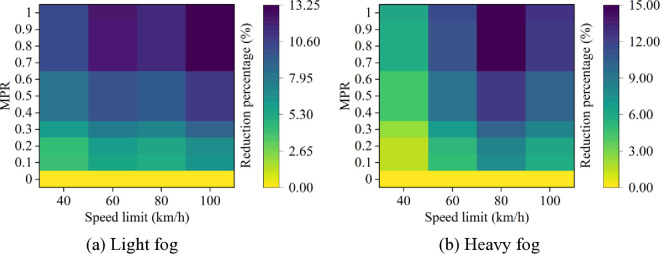
NO_x_ emissions reduction percentage under different conditions.

Based on these results from Figs 7–11, it is evident that as MPR increases, fuel consumption and emissions decrease, indicating that the proposed car-following strategy of CAVs effectively reduces fuel consumption and emissions in foggy conditions. Using MPR = 0 as the baseline, the reduction ratios for fuel consumption and emissions across different MPR conditions are calculated and presented in Figs 12–16. It is clear that with an increase in MPR, the reduction ratios of fuel consumption and emissions increases across all scenarios. When MPR = 1, the average reductions for fuel consumption, CO_2_ emissions, CO emissions, HC emissions, and NOx emissions across different scenarios are 27.62%, 27.62%, 9.57%, 6.21%, and 11.55%, respectively.

Furthermore, it can be observed that the highest reduction ratios for fuel consumption and emissions occur under light and heavy fog scenarios when speed limit is 100 km/h and 80 km/h, respectively, while the lowest reduction ratios are calculated when speed limit is 40 km/h. This suggests that the greater the fuel consumption and emissions in the baseline scenario, the more significant the reduction effect of CAVs in those conditions. Similar to the car-following risk reduction, the increase in reduction ratios for fuel consumption and emissions is rapid when MPR < 0.6. However, when MPR ≥ 0.6, the rate of increase in reduction ratios slows down.

**Fig 17:**
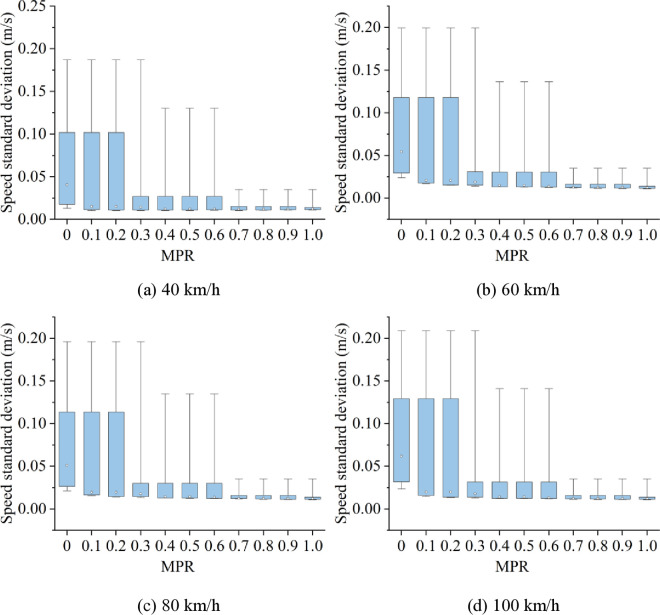
The speed standard deviation in light fog under various speed limits.

**Fig 18:**
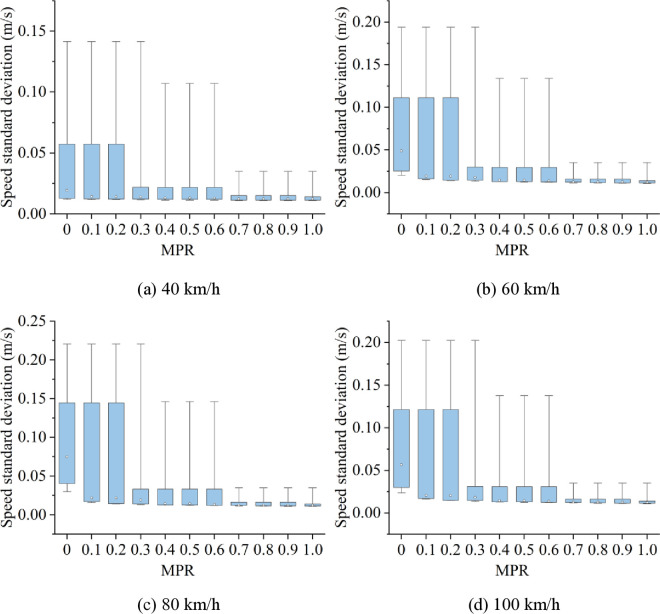
The speed standard deviation in heavy fog under various speed limits.

Related studies have shown that car-following risk and energy consumption are intrinsically related to speed fluctuations. To explore the underlying reasons for the changes in car-following risk and energy consumption under different scenarios, as well as the effectiveness of CAVs in mitigating these characteristics, speed standard deviation in various scenarios is calculated, as shown in Figs 17 and 18.

Based on Figs 17 and 18, it is discovered that when MPR = 0, speed standard deviation is highest in light and heavy fog scenarios when speed limits are expressed as 100 km/h and 80 km/h, respectively, and lowest while speed limit is 40 km/h. In scenarios with speed limits of 40, 60, and 100 km/h, standard deviation of speeds in light fog scenario is higher than in the heavy fog scenario, whereas, the speed standard deviation in light fog scenario is lower than that in the heavy fog scenario under speed limit maintains 80 km/h. This indicates that, under different fog densities and speed limit conditions, the speed standard deviation follows a similar trend to car-following risk and energy consumption.

Since the speed standard deviation reflects the trend of speed fluctuations from a statistical perspective, we can conclude from the above analysis that car-following risk and energy consumption on foggy highways are closely related to speed fluctuations. The greater the speed fluctuations, the higher the car-following risk and energy consumption. Furthermore, as MPR increases, the speed standard deviation decreases in all scenarios. Therefore, the proposed CAV control strategy can reduce speed fluctuations, thereby lowering car-following risk and energy consumption including fuel consumption and traffic emissions.

Furthermore, from Fig 17 and Fig 18, we can find that as the MPR increases, the standard deviation of speed initially decreases rapidly, followed by a more gradual decline. The inflection point of this trend also occurs at MPR = 0.6, further confirming that the reduction in speed fluctuations is a key factor driving the observed decreases in car-following risk and energy consumption. The explanation for this phenomenon is as follows: When the MPR of CAVs is below 0.6, increasing the number of CAVs significantly suppresses the propagation of speed fluctuations within the vehicle platoon. Consequently, reductions in energy consumption and car-following risk occur rapidly. However, when MPR reaches or exceeds 0.6, the platoon becomes predominantly composed of CAVs, and vehicle coordination approaches an optimal state. At this stage, speed fluctuations generated by the lead vehicle are effectively dampened by the CAVs in the platoon, resulting in a stabilized traffic flow. As a result, the marginal benefits of further increasing the CAV proportion in terms of reducing energy consumption and car-following risk diminish, and the downward trend slows accordingly. This leads to the conclusion that the decreasing trends of car-following risk and energy consumption are consistent with the reduction in speed standard deviation, with all exhibiting the same inflection point.

This study presents a MPC-based CAV control strategy in foggy conditions, aiming to reduce car-following collision risks and energy consumption. To accomplish this objective, the IDM model is initially chosen, which is fine-tuned based on driving simulator data, to characterize HDVs’ car-following dynamics under foggy weather scenarios. We then present a CAVs control strategy. Finally, to verify the efficacy of the proposed CAV control strategy in mitigating car-following risks and energy consumption, a series of numerical experiments are carried out, and a systematic analysis is performed on car-following risks and energy consumption under different CAV MPRs conditions.

This study draws the following conclusions. Under different fog densities and speed limits, car-following risks and energy consumption vary. Under light and heavy fog conditions, the highest values of ITC and DRAC occur at a 60 km/h speed limit and 80 km/h speed limit, respectively, while the lowest values are observed at a speed limit of 40 km/h. Similarly, in light fog and heavy fog, the highest energy consumption occurs at speed limits of 100 km/h and 80 km/h, respectively, while the lowest emissions are found at 40 km/h. The developed control strategy notably reduces both car-following risks and energy consumption. Both car-following risks and energy consumption decrease with an increase in MPR. When MPR = 1, the average reduction in ITC, DRAC, fuel consumption, CO_2_ emissions, CO emissions, HC emissions, and NOx emissions across different scenarios is 80.74%, 59.44%, 27.62%, 27.62%, 9.57%, 6.21%, and 11.55%, respectively. Additionally, when MPR < 0.6, the reductions in car-following risks and energy consumption are rapid, while the reductions become slower when CAV MPR exceeds 0.6.

The primary contribution of this paper lies in examining the impact of different fog densities and speed limits on car-following risks and energy consumption/emissions from the vehicle operation perspective. Furthermore, it proposes a CAVs control strategy capable of significantly reducing both car-following risks and energy consumption/emissions. Through simulation experiments, we observed that speed fluctuations vary under different fog densities and speed limits, and there is an inherent relationship between speed fluctuations and both car-following risks and energy consumption/emissions. This results in differing impacts of fog density and speed limits on these factors. Moreover, in scenarios with larger speed fluctuations, car-following risks and energy consumption/emissions are higher. The proposed control strategy can improve vehicle operations in foggy environments, reduce speed fluctuations, and thus lower both car-following risks and energy consumption/emissions.

The findings of this study provide insights for speed limit and dedicated lane management in foggy conditions. As analyzed in Sections 4.2 and 4.3, when MPR = 0, both light and dense fog scenarios exhibit higher car-following risks and energy consumption at speed limits of 100 km/h and 80 km/h, respectively, compared to the higher risks and emissions observed at a 40 km/h limit. Additionally, at the 40 km/h speed limit, the standard deviation of speed is the smallest among all speed conditions, indicating improved acceleration and deceleration behavior within the platoon. This suggests that a 40 km/h speed limit contributes to the stability of the entire traffic flow, offering valuable guidance for speed limit management on highways in foggy conditions. Before the full deployment of CAVs on highways, a 40 km/h speed limit should be implemented in both light and dense fog scenarios, while the 100 km/h and 80 km/h limits should be avoided to suppress the transmission of speed fluctuations, reduce frequent acceleration and deceleration, and consequently lower car-following risk and energy consumption. Similarly, when MPR = 1, both light and dense fog scenarios show higher car-following risks and energy consumption at a 40 km/h speed limit but lower risks and emissions at 100 km/h and 60 km/h, respectively. Therefore, upon full deployment of CAVs, speed limits of 100 km/h and 60 km/h should be applied in light and dense fog conditions, while the 40 km/h limit should be avoided. For mixed traffic flow in foggy conditions, speed limits should be adjusted flexibly based on the relative levels of car-following risk and energy consumption under different MPR and speed limit scenarios.

Moreover, based on the analysis in Sections 4.2 and 4.3, an MPR of 0.6 represents the inflection point in the curves for the reduction of car-following risk and energy consumption. This finding provides valuable guidance for managing mixed traffic flow and dedicated lane allocation on highways under low MPR conditions in foggy weather. Specifically, future highways could have two types of lanes: one for mixed traffic flow, consisting of both CAVs and HDVs, and another dedicated exclusively to CAVs. When the MPR in the mixed flow lane exceeds 0.6, the analysis reveals that further increasing the number of CAVs in the platoon will not significantly reduce car-following risk and energy consumption. Therefore, it is necessary to limit the number of CAVs entering the platoon, ensuring that the minimum number of CAVs achieves the maximum reduction in car-following risk and energy consumption in the mixed traffic flow. This would allow CAVs to fully exploit their potential in reducing risks and emissions, while excess CAVs could be directed to the dedicated CAV lane. In the CAV-only lane, where there is no interference from HDVs, CAVs can fully utilize their advantages, improving both the reduction in car-following risk and energy consumption while enhancing overall traffic flow efficiency.

This study also has some limitations. The simulated scenarios designed in this study do not account for lateral lane change modeling. On real highways, lateral lane change behavior and longitudinal following behavior often coexist. Therefore, more complex and realistic simulation scenarios should be designed to assess the car-following collision risks and energy consumption under foggy conditions more comprehensively.

## Supporting information

S1 Data Data. (XLSX)
